# Massachusetts Veterinary Medical Society

**Published:** 1891-08

**Authors:** Austin Peters

**Affiliations:** Secretary


### SOCIETY PROCEEDINGS.

*Massachusetts Veterinary Association.*—The regular meeting of the Massachusetts Veterinary Association was held at 19 Boylston Place, Boston, Wednesday evening, June 24th, 1891. President L. H. Howard in the chair. Members present: Drs. Beckett, Blackwood, Emerson, Haddock, Howard, Marshall, Winslow and the Secretary. Honorary Member Dr. Stickney.

Minutes of the previous meeting read and accepted.

Unfinished business. The Secretary reported having seen President Goodell, of the Mass. Agricultural College, at Amherst, at commencement, with reference to the appointment of a veterinary scientist upon the staff of the State Experiment Station. Dr. Winchester had also talked with President Goodell about it at the same time. Both President Goodell and Professor Goessmann were in favor of such an appointment, and the Board of Control, of the Experiment Station, at the annual meeting, held at Amherst, June 9th, had appointed a committee, consisting of President Goodell and Secretary Sessions, of the State Board of Agriculture, to meet a similar committee from the Massachusetts Veterinary Association, to discuss the proper steps to be taken toward the establishment of a veterinary department at the State Experiment Station.

Dr. Stickney spoke in favor of the Association's taking action to bring about such a state of affairs. Dr. Blackwood suggested that the chair might appoint a committee to meet the committee from the Board of Control, of the Experiment Station. Dr. Marshall then moved that the chair appoint a committee of two to confer with President Goodell and Secretary Sessions. Seconded by Dr. Blackwood and carried.

The President then named Drs. Winchester and Peters to serve on the committee.

The Secretary then reported that he had taken no steps towards notifying all veterinary graduates in the State of the existence of the Association, or towards having copies of the constitution printed, because he was instructed to notify *all* veterinary graduates, and there were some he thought it would be as well not to notify. And with regard to the constitution, he thought it might be well to modify it by doing away with the clause requiring a thesis from applicants for membership as one of the conditions of admission.

After considerable discussion it was decided that the matter of notifying veterinary graduates of the existence and objects of the Association could safely be left at the discretion of the Secretary.

A request in writing was then filed with the Secretary that Article II, section *a*, of the constitution, be amended so that section *a* of said Article be rendered inoperative for a period of two years from April, 1891, i.e., that the thesis as a requirement for admission be not called for during the period named.

Under the provisions of the constitution governing amendments no action can be taken until the next regular meeting, which does not occur until September.

New business : The work done at the session of the Legislature, just adjourned, for recognition and advancement of veterinary science, by one of its members, Mr. F. H. Appleton, of Peabody, was then brought up.

Dr. Marshall moved that the Secretary be instructed to extend a formal vote of thanks from the Massachusetts Veterinary Association to Francis H. Appleton, Esq., for the kind and friendly interest he had shown in the welfare of a hitherto rather neglected profession.

Seconded by Dr. Hadcock. Carried unanimously.

Meeting then adjourned.

AUSTIN PETERS, *Secretary.*

